# The Mystery Actor in the Neuroendocrine Theater: Who Really Knows Obestatin? Central Focus on Hypothalamic–Pituitary Axes

**DOI:** 10.3390/ijms26157395

**Published:** 2025-07-31

**Authors:** Michał Szlis, Anna Wójcik-Gładysz, Alina Gajewska, Bartosz Jaroslaw Przybyl

**Affiliations:** Department of Animal Physiology, The Kielanowski Institute of Animal Physiology and Nutrition, Polish Academy of Sciences, Instytucka 3, 05-110 Jabłonna, Poland; m.szlis@ifzz.pl (M.S.); a.wojcik@ifzz.pl (A.W.-G.); a.gajewska@ifzz.pl (A.G.)

**Keywords:** obestatin, central nervous system, somatotrophic axis, gonadotrophic axis

## Abstract

The available literature data indicate that obestatin, a peptide derived from the preproghrelin precursor, may modulate neuroendocrine function, particularly in appetite regulation and somatotrophic/gonadotrophic pathways. This review synthesizes animal studies assessing the influence of obestatin on central neuroendocrine systems. Obestatin has been shown to affect the hypothalamic appetite-regulating center through neuropeptides such as neuropeptide Y and agouti-related peptide, yet findings remain inconsistent between species. In rodents, its effects on food intake and energy balance are inconclusive, whereas sheep models demonstrate significant alterations in orexigenic gene expression and peptide immunoreactivity. Regarding the somatotrophic axis, obestatin showed no significant effect on growth hormone (GH) secretion in rodents; however, in sheep, it modulated growth hormone-releasing hormone and somatostatin mRNA expression, elevated pituitary GH synthesis, and increased circulating GH levels. Studies involving the gonadotrophic axis demonstrated the presence of obestatin in Leydig and pituitary cells, with in vitro evidence suggesting its ability to modulate intracellular pathways implicated in gonadoliberin, luteinizing hormone, and follicle-stimulating hormone release. The collective findings discussed in this article indicate that obestatin interacts with multiple hypothalamic–pituitary axes, though its effects vary depending on species and experimental conditions. This review highlights the complexity of obestatin’s central actions and the need for further research to elucidate its functional relevance in neuroendocrine regulation.

## 1. Introduction

Both animals and humans live in a diverse and constantly changing environment that generates a vast array of stimuli. Maintaining a state of relative physiological balance is necessary for the proper functioning of all physiological processes. The integration and processing of incoming stimuli occurs in the central nervous system (CNS), which relays information to other organs and tissues. Effective coordination of functions at different levels relies on a communication network involving the circulatory, nervous, and endocrine pathways.

Availability of energy from the external environment is one of the crucial factors influencing proper metabolism, thermogenesis, the rate of cellular metabolism, as well as the development and function of the nervous system. Both short-term and chronic food deficiency result in the inhibition of the thyroid axis secretory activity, which consequently may lead to significant clinical consequences, including reduced metabolic processes, cardiac arrhythmias, reproductive abnormalities, and psychiatric symptoms such as depression or anxiety. Furthermore, controlling reproductive and growth processes is important, not only from the individual point of view but also from the maintenance of species. Reproduction, especially in females, is an energy-requiring process, so the relationship between the reproductive system functioning and the organism’s energy status is an important aspect. Both short-term and chronic food deficiency result in the inhibition of gonadotrophic and somatotrophic axes activity, which consequently leads to a lower number of offspring, temporary infertility, or stunted growth of the body. It is noteworthy that the activity of those axes is conditioned by a number of metabolic and nutritional signals, suggesting that common regulatory pathways are involved in the joint control of reproduction, growth, and energy balance [1].

New compounds, including hormones and neurotransmitters, continue to be discovered, and further studies confirm their involvement in the regulation of key physiological processes. One of them is obestatin, a 23-amino-acid (aa) peptide (2.5 kDa) encoded by the GHRL gene, whose translation produces a preproghrelin precursor protein. Subsequently, both ghrelin and obestatin peptides are generated through post-translational cleavage of preproghrelin [2]. Initial studies proposed an anorexigenic role for obestatin, demonstrating its ability to suppress food intake, reduce body weight, and delay gastric emptying [2,3,4]. However, subsequent reports presented varying results depending on obestatin administration route and dosage [5,6]. Obestatin was originally isolated from rat stomach mucosa cells [2], but subsequent studies showed that its synthesis was not limited to this organ. Huda et al. (2008) [7] demonstrated that blood obestatin levels remained stable after gastrectomy, confirming its production in other tissues. Immunohistochemical analyses have localized obestatin to multiple organs, including the duodenum, jejunum, large intestine, pancreas, liver, mammary glands, testicles (Leydig cells), and lungs, as well as in body fluids such as saliva and blood plasma [2,7,8,9].

The GHRL gene, encoding obestatin, is located on chromosome 3p26-p25 in humans and chromosome 4q4 in rats. The gene consists of four exons, three introns, and an additional small (20 bp) exon 0, responsible for encoding the 117 aa preproghrelin precursor [10,11]. Recent studies identified an additional exon (−1) and demonstrated that alternative splicing of exons −1, 3, and 4 generates functional obestatin [12,13,14]. Studies on the properties of obestatin initially indicated its affinity for the orphan G protein-coupled receptor 39 (GPR39) [2]. This receptor, a member of the rhodopsin-like receptor family, was first cloned by [15] as part of a family related to growth hormone secretagogue receptors (GHS-R). Analysis of GPR39 expression revealed the presence of two isoforms: the full-length GPR39-1a (423 aa), highly expressed in peripheral metabolic tissues (liver, pancreas, kidneys, adipose tissue), and the truncated GPR39-1b (284 aa), predominantly localized to the central nervous system [16]. However, subsequent GPR39 knockout mouse models showed no functional response to obestatin [17,18,19], challenging its role as the canonical obestatin receptor.

A study by Granata et al., (2008) [20] indicated potential obestatin binding to the glucagon-like peptide type 1 receptor (GLP-1R), involved in appetite regulation in the CNS; however, subsequent research by Unniappan et al., (2008) [21] failed to replicate these findings. These discrepancies in available data may result from the unresolved identification of the receptor and the limited information on specific agonists and antagonists of this peptide. Therefore, further research is necessary to identify the obestatin receptor and determine its location. Similar to ghrelin, obestatin appears capable of crossing the blood–brain barrier. Ref. [22] demonstrated this property through intravenous administration of radiolabeled obestatin, which showed both rapid brain uptake and concurrent metabolic degradation. This phenomenon is likely due to the short half-life of obestatin in blood plasma, estimated at approximately 22 min [22].

## 2. Neurohormonal Regulatory Networks

The hypothalamus integrates signals from both the body’s periphery and external environment. This small structure is limited by the ventral part of the lateral and ventral walls of the third ventricle of the brain, located between the optic chiasm anteriorly and the mammillary bodies posteriorly [23]. Its neuroanatomical organization comprises distinct clusters of neuronal cell bodies (perikarya) called hypothalamic nuclei. These nuclei and their neural projections form interconnected networks that regulate fundamental physiological processes, including body weight control, energy homeostasis, thermoregulation, reproduction, and growth.

**Figure 1:**
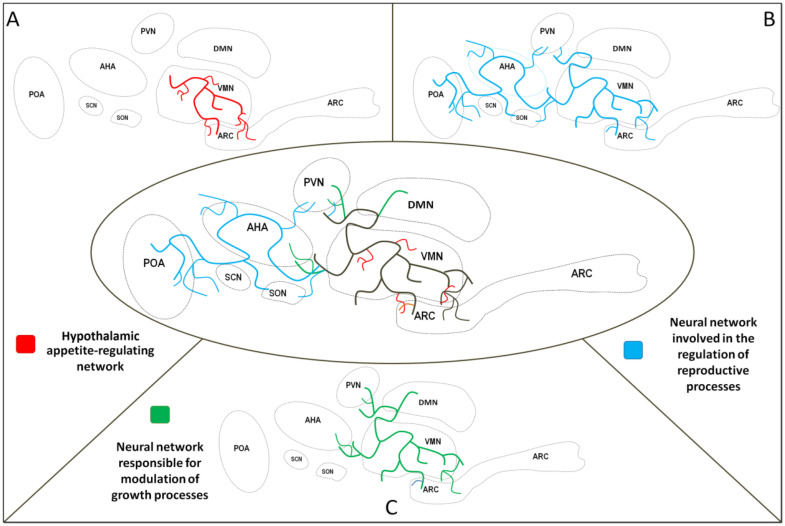
Selected neural networks in the hypothalamus. Shared area of neural networks—black lines; neural network involved in the regulation of energy homeostasis—red lines (**A**). Neural network involved in the regulation of reproductive processes—blue lines (**B**). Neural network responsible for the modulation of growth processes—green lines (**C**). PVN—paraventricular nucleus, VMN—ventromedial nucleus, DMN—dorsomedial nucleus, ARC—arcuate nucleus, POA—preoptic area, AHA—anterior hypothalamic area, SCN—suprachiasmatic nucleus, SON—supraoptic nucleus.

Key hypothalamic nuclei include the paraventricular nucleus (PVN), suprachiasmatic nucleus (SCN), supraoptic nucleus (SON), ventromedial nucleus (VMN), dorsomedial nucleus (DMN), and arcuate nucleus (ARC). While these nuclei were initially considered to independently regulate distinct physiological processes, current evidence demonstrates significant functional overlap. Neuroanatomical studies demonstrate that brain areas governing food intake regulation substantially coincide with those controlling growth and reproductive processes (Figure 1). Furthermore, key nuclei comprising the appetite-regulating network, including the ARC, PVN, VMN, and DMN, colocalize with the area responsible for the synthesis of key neurohormones that modulate growth and reproduction [24,25]. The release of hypothalamic neuropeptides into the hypothalamic–pituitary portal circulation system occurs in the median eminence (ME). This structure is a periventricular organ composed of blood vessels from the hypothalamic–pituitary portal circulation system and nerve terminals from hypothalamic nuclei and other brain regions. Through this unique vascular-neural interface, the hypothalamus regulates pituitary secretion of key hormones, including those of the somatotrophic axis that control body growth and the gonadotrophic axis governing reproductive functions.

Numerous studies have demonstrated that the body’s energy status is a key factor in the proper functioning of the somatotrophic and gonadotrophic axes [26,27]. Both insufficient energy (malnutrition, nutrient deficiencies) and excess energy states (overweight, obesity) disrupt these endocrine systems. Ongoing research in many centers worldwide aims to clarify the mechanisms that link the body’s energy status with the regulation of growth and reproductive functions.

## 3. Effect of Obestatin on Neurohormonal Network Activity

### 3.1. Obestatin’s Influence on Body Energy Status/Appetite-Regulating Network

The mediobasal hypothalamus (MBH) contains a few specialized neuronal populations that play a critical role in metabolic regulation and energy homeostasis [28]. Among others, two main neuronal subpopulations have been identified in the ARC: the first coexpresses neuropeptide Y (NPY) and agouti-related peptide (AgRP; NPY/AgRP neurons), while the second coexpresses cocaine- and amphetamine-regulated transcript (CART) and α-melanotropin (α-MSH; CART/α-MSH neurons). These neurons send their axonal projections to multiple hypothalamic regions, including the PVN, VMN, POA, and AHA [29,30,31,32], and are involved in the regulation of food intake and energy expenditure [33,34].

NPY represents one of the most abundant peptides in the brain, with its primary hypothalamic synthesis occurring in the ARC [35]. Recognized as one of the most potent orexigenic factors, NPY strongly stimulates appetite and food intake through its actions on Y1 and Y5 receptors, which belong to the G protein-coupled receptor family [36,37]. The expression of NPY is dynamically regulated by the body’s energy status: under conditions of optimal nutrition, the amount of NPY in nerve cells decreases, while starvation and/or malnutrition elevate its secretion [26,38,39,40]. AgRP also exerts potent orexigenic effects, though unlike NPY, its expression appears restricted to the ARC [41]. Similar to NPY, AgRP expression is dynamically regulated by energy status. Central administration of AgRP stimulates hyperphagia while reducing energy consumption, ultimately causing weight gain. Mechanistically, AgRP functions as an endogenous antagonist of melanocortin receptors MC3R and MC4R, counteracting the appetite-suppressing effects of α-MSH [39,42].

The α-MSH peptide is produced through post-translational processing of the precursor protein pro-opiomelanocortin (POMC). In contrast to the previously described peptides, α-MSH is an anorexigenic hormone that reduces food intake [43] through activation of melanocortin receptors MC3R and MC4R, which are predominantly expressed in the PVN [44]. As with NPY and AgRP, the expression of the *Pomc* gene and α-MSH protein is also associated with the nutritional status of the organism: starvation decreases α-MSH levels, while refeeding increases its production [45]. CART is another extremely important peptide associated with the regulation of energy status in ARC neurons [46]. This peptide exhibits anorexigenic properties similar to α-MSH. Studies show that fasting reduces CART secretion, while restored food intake stimulates its release [47,48,49]. Although no specific CART receptor has been identified, in vitro evidence suggests interactions with G protein-coupled receptors [50].

Both NPY/AgRP and CART/α-MSH neurons express receptors for several peripheral peptides and proteins involved in energy regulation, including leptin and ghrelin [42]. Moreover, direct connections have been identified between these neuronal populations and neurons responsible for the secretion of SOM, GHRH, and GnRH hormones. This suggests that NPY/AgRP and CART/α-MSH neurons, particularly NPY neurons, play a central role in transmitting information about energy status and integrating it with the functioning of the somatotrophic and gonadotrophic axes.

**Figure 2:**
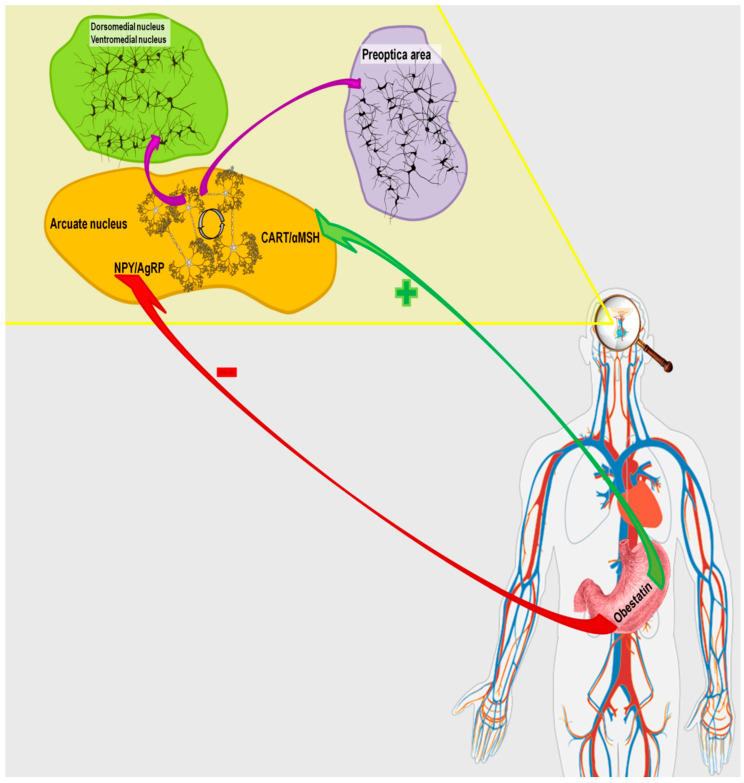
Pathway of obestatin action in the hypothalamic network regulating the body’s energy homeostasis.

The literature data indicate that obestatin may modulate the activity of the hypothalamic appetite-regulating network, but the outcomes vary depending on the species (Figure 2). Studies in rats demonstrated that obestatin induces appetite suppression, body weight reduction, and delays gastric emptying [2]. Surprisingly, subsequent investigations in rodents found no significant effect of peripherally or centrally administered obestatin on food intake, energy expenditure, or body weight [51,52]. In addition, research on intraventricular administration of obestatin in rats showed no changes in hypothalamic mRNA expression of NPY and AgRP [4]. In sheep, intracerebroventricular administration of obestatin increased mRNA expression of NPY, AGRP, and NPY1R in MBH neurons. On the other hand, the same study reported decreased NPY immunoreactivity in ARC nucleus perikarya and nerve fibers, along with reduced NPY nerve fiber density in the PEV nucleus [53].

### 3.2. Obestatin and Somatotrophic Axis

The hypothalamic regions involved in regulating the somatotrophic axis include the anterior hypothalamic area (AHA) as well as the PVN, DMN, VMN, and ARC. This axis (hypothalamus–pituitary–peripheral tissue cells) is primarily controlled by two antagonistic neurohormones: somatostatin (SRIF) and growth-hormone-releasing hormone (GHRH). The antagonistic interplay between GHRH and SRIF results in the pulsatile release of growth hormone (GH) from pituitary somatotrophic cells into circulation [40,54,55,56]. The direct effects of SRIF and GHRH on pituitary somatotrophs are mediated by their specific receptors: GHRH receptor increases intracellular cAMP levels, triggering signaling pathways that enhance GH gene expression, while SRIF receptor inhibits GH production through cAMP suppression [57].

Growth hormone is a major regulator of peripheral growth processes. This 23 kDa polypeptide exhibits high interspecies sequence and structural homology, reflecting its fundamental biological role [58]. Despite this homology, GH half-life varies between species, from approximately 6 min in rats to about 25 min in humans [59,60]. Pulsatile GH secretion from pituitary somatotrophs results from alternating SRIF and GHRH release at the median eminence. GH exerts its effects on peripheral tissues either directly or indirectly through insulin-like growth factors (IGF) type 1 and 2 (IGF-1, IGF-2) [61,62]. The main effect of GH, mediated by IGF-1 and IGF-2, is the stimulation of body mass gain by promoting chondrogenesis and osteogenesis in bone cartilage. GH also directly regulates carbohydrate metabolism by inducing hepatic glycogenolysis and increasing glucose release. In adipose tissue, GH enhances lipolysis and suppresses lipogenesis, ultimately elevating plasma free fatty acid concentrations.

**Figure 3:**
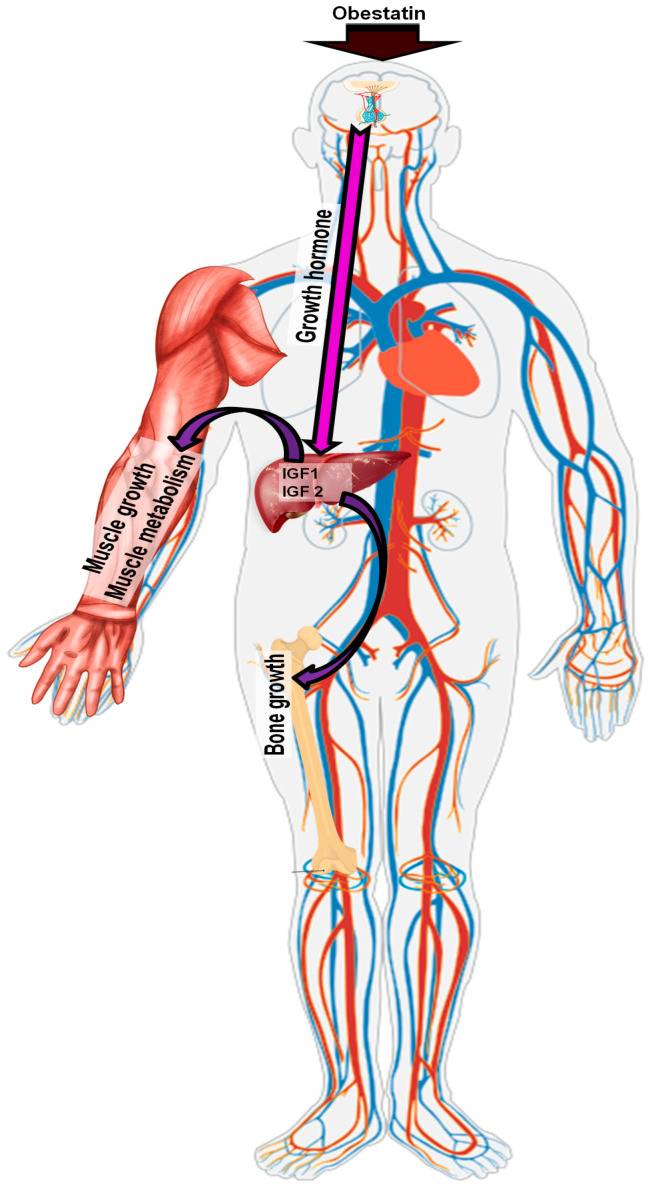
Pathway of obestatin action in the somatotrophic axis.

Current understanding of obestatin’s effects on GH regulation is primarily based on rodent studies (Figure 3). Initial research focused on explaining the potential effect of obestatin on GH secretion from pituitary somatotrophic cells [2]. In vivo studies in rats showed no effect of obestatin on either spontaneous or ghrelin-induced GH release [63]. Similarly, intraperitoneal administration of obestatin did not affect the change in GH levels in 10-day-old rats [5]. Moreover, Zizzari et al. (2007) [64] demonstrated that obestatin antagonizes ghrelin-dependent GH release in vivo. In vitro studies performed on rat cell lines or pituitary explants showed no effect of obestatin on either spontaneous or ghrelin-dependent GH release [2,64]. Contrasting findings were reported by Pazos et al., (2009) [65], who demonstrated obestatin-induced GH release in GC cell lines derived from mouse somatotrophic tumors.

Recent research has identified a potential role of obestatin in the hypothalamus. Specifically, experiments on mouse hypothalamus explants have demonstrated that obestatin inhibits ghrelin-dependent GHRH release, while not altering the activity of SOM neurons [66,67]. Food restrictions in mice and rats cause reduced secretory activity of somatotrophic pituitary cells and a significant decrease in blood GH levels. In ruminants, however, similar conditions result in increased GH synthesis and release [68]. These changes are largely attributed to the removal of the inhibitory effect of SOM [26,69]. Nevertheless, it should be noted that in ruminants, the mechanism regulating growth processes differs from that in rodents, which are monogastric animals with distinct eating habits and gastrointestinal physiology. A study on sheep subjected to intraventricular administration of obestatin to the third ventricle of the brain demonstrated that this peptide could regulate somatotrophic axis activity at the hypothalamic level. Intraventricular obestatin administration inhibited SOM mRNA expression in both the AHA and ME, while simultaneously stimulating GHRH transcription in the MBH. These hypothalamic effects were accompanied by significant endocrine changes, including increased GH mRNA production, elevated numbers of GH-immunoreactive cells in the pituitary, and higher circulating GH levels characterized by enhanced pulse frequency (Table 1) [6].

### 3.3. Obestatin and Gonadotrophic Axis

Gonadoliberin (GnRH), produced primarily in POA neurons, serves as the key regulator of the gonadotrophic axis. In sheep, approximately 70% of GnRH neurons project to the median eminence inner layer [70], where the peptide is released into the hypothalamic–pituitary portal circulation to control pituitary gonadotropin secretion. GnRH is released in a pulsatile manner, mainly controlled by the GnRH pulse generator system, which includes ARC neurons coexpressing kisspeptin (Kiss), neurokinin B (NKB), and dynorphin (Dyn)—collectively termed KNDy neurons [71]. Increased Kiss release from KNDy neuron terminals in the ME stimulates GnRH secretion into the local blood vessels [72]. Kiss release itself is modulated through auto- and paracrine mechanisms by NKB and Dyn [73]. Specifically, NKB stimulates the synthesis and release of Kiss, whereas Dyn inhibits these processes [71]. GnRH acts directly on pituitary gonadotrophs through specific G protein-coupled receptors (GnRHR). While multiple GnRHR types have been identified in various animal taxa, they all belong to the G protein-coupled receptor family [74], with GnRH receptor type I (GnRH1R) and type II receptor (GnRH2R) being the main receptors in mammals [75,76,77]. GnRHR activation primarily stimulates luteinizing hormone (LH) secretion by pituitary gonadotrophs, with secondary stimulation of follicle-stimulating hormone (FSH) release [78].

LH secretion occurs in a pulsatile pattern directly following GnRH pulses. In females, LH regulates estrogen production, influencing the development of the corpus luteum, progesterone synthesis, and ovulation. In males, LH is responsible for testosterone secretion and the development of secondary sexual traits (according to Pierzchała-Koziec, 2005 [79]). Meanwhile, FSH regulation involves two distinct mechanisms: a constitutive pathway that operates independently of GnRH pulses and a pulse-dependent pathway. The complexity of these two mechanisms may be additionally modulated by locally acting pituitary factors, including inhibins, activins, or follistatins, which form autocrine/paracrine regulatory networks for FSH control [78,80].

**Figure 4:**
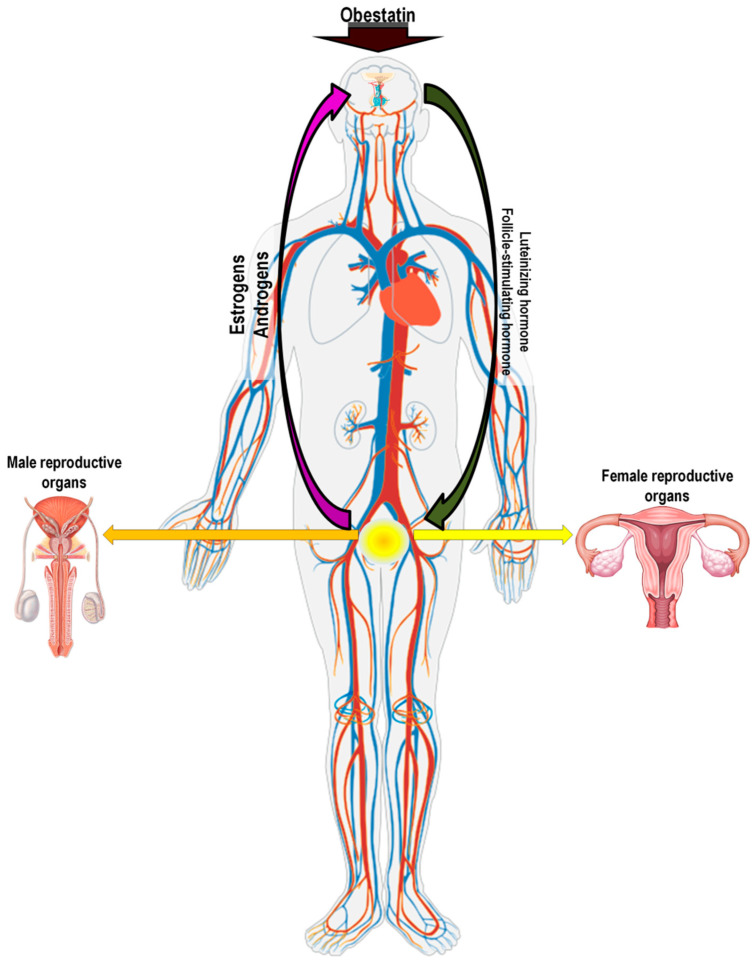
Pathway of obestatin action in the gonadotrophic axis.

Current research has demonstrated that obestatin is produced in the male gonads by Leydig cells and is also present in pituitary cells [9,81]. In vitro studies on mouse, rat, and porcine cell lines have revealed that obestatin stimulates the secretion of both cAMP and the cellular signaling protein ERK1/2, while inhibiting myogen-activated kinase (MAPK). Additionally, the increase in cAMP has been associated with the activation of the cellular cAMP/protein kinase A-dependent pathway (PKA; [2,82,83,84]). The cAMP/PKA pathway, ERK1/2, and MAPK are known to mediate numerous hormonal signals, including intracellular signaling pathways involved in the synthesis and secretion of GnRH, LH, and FSH [83,85,86]. In vitro studies in porcine granulosa cells showed that obestatin stimulated progesterone secretion into the culture medium, without affecting estradiol and testosterone secretion [83]. However, contrasting results were obtained in human luteal cells, where obestatin reduced progesterone and prostaglandin (E2 and F2α) secretion [87]. Other in vitro studies using mouse cell lines, as well as in vivo experiments in mice, found no effect of obestatin on prolactin, LH, or FSH release from pituitary gonadotropes (Figure 4) [88].

**Table 1:** The influence of obestatin on the somatotrophic axis.

Authors	Research Model	Materials and Methods	Outcome
Feng et al., 2011 [66]	Male mice	Incubation of hypothalamic explants	No effect of obestatin on spontaneous and ghrelin-induced reduction in somatostatin release from hypothalamic nerve cells. No effect on the activity of GHRH neurons and blocking ghrelin-induced GHRH release.
Hassouna et al., 2012 [67]	Male mice	Intraperitoneal injection in vivo	Obestatin abolished the stimulatory effect of ghrelin on the activity of GHRH neurons. Reduction in GH concentration as a result of obestatin eliminating the stimulatory effect of ghrelin.
Nogueiras et al., 2007 [4]	Male rats	Intravenous administration	Obestatin does not affect the release of GH into the blood.
Yamamoto et al., 2007 [63]	Male rats	Intravenous/intracerebroventricular administration	Obestatin does not affect the release of GH into the blood.
Bresciani et al., 2006 [5]	Male rats	Intraperitoneal injection	Obestatin does not affect the release of GH into the blood.
Zizzari et al., 2007 [64]	Male mice and rats	Intraperitoneal injection/intravenous injection	Obestatin antagonizes ghrelin-dependent GH release in vitro.
Zhang et al., 2005 [2]	Male rats	48 h fasting	No effect of obestatin on spontaneous and ghrelin-dependent GH release.
Pazos et al., 2009 [65]	GC cells line	Medium supplemented with obestatin	Obestatin stimulated GH release into the culture medium, but only within the first 15 min of incubation.
Luque et al., 2014 [88]	Pituitary cells from baboons and mice	Medium supplemented with obestatin	24 h incubation of cells in medium supplemented with obestatin resulted in decreased GH mRNA expression and reduced GH release.
Wójcik-Gładysz et al., 2018 [6]	Female sheep	Intracerebroventricular infusions	The mechanism of obestatin action seems to rely simultaneously on the stimulation of GHRH and the restraint of somatostatin output, which results in the enhanced release of GH from pituitary somatotrophic cells to the peripheral circulation.

Research in sheep has revealed that obestatin exerts multi-level control over reproductive neuroendocrine function. When administered intracerebroventricularly, obestatin displayed region-specific regulation of GnRH neurons, stimulating GnRH mRNA expression in the POA, while suppressing it in the ME. This was accompanied by altered GnRH peptide distribution in ME nerve terminals, suggesting effects on both synthesis and release mechanisms. The peptide also modified GnRH pulse generator activity, indicating direct neuromodulatory actions on GnRH neuronal networks. Additionally, obestatin treatment triggered opposing effects in key neuropeptide systems by upregulating kisspeptin (Kiss) mRNA while downregulating prodynorphin (pDyn) expression. Paradoxically, despite increased kisspeptin transcription, immunohistochemical analysis revealed decreased kisspeptin immunoreactivity in both ARC perikarya and ME nerve fibers. These changes in GnRH regulation were further supported by subsequent research examining obestatin and its effects on LH and FSH release into the bloodstream. Obestatin decreased LHβ mRNA expression while increasing LH accumulation in gonadotropic cells, ultimately reducing circulating LH concentrations. Interestingly, the same study reported an increase in both FSHβ mRNA expression and FSH immunoreactivity in pituitary cells, although the mean blood FSH concentration remained unchanged (Table 2) [89,90].

## 4. Summary and Future Research Directions

The physiological role of obestatin, a peptide hormone derived from the same precursor as ghrelin, remains complex and incompletely understood. Findings related to the somatotrophic axis activity suggest a limited or inhibitory role of obestatin in GH regulation in monogastric animals, such as rodents, based on both in vivo and in vitro studies. In contrast, results in ruminants, especially sheep, reveal a remarkably different pattern. When administered directly to the brain, obestatin inhibits somatostatin mRNA expression, simultaneously increasing GHRH transcript levels, leading to elevated pituitary GH synthesis and secretion into peripheral blood. Obestatin also appears to influence the activity of the hypothalamic–pituitary–gonadal axis. While in vitro studies have reported varied effects on steroid hormone release, in vivo sheep experiments indicated that obestatin modulates LH and FSH expression and secretion. Additionally, obestatin affects the GnRH pulse generator, thereby regulating GnRH neuronal activity. Furthermore, obestatin appears to interact with the hypothalamic appetite-regulating network, although its effects show species-specific variation. In rodents, obestatin may suppress appetite, whereas in sheep, it upregulates the expression of orexigenic peptides (NPY and AgRP).

The current literature lacks information on the potential therapeutic effects of obestatin in the neuroendocrine systems discussed in this review, namely the appetite regulation system and the somatotrophic and gonadotrophic axes. Nonetheless, there are some findings that suggest this peptide may be involved in therapeutic applications across various physiological systems. In a study conducted on rats with type 2 diabetes, it was demonstrated that obestatin administered over a period of 30 days had a modulatory effect on blood insulin and glucose concentrations. These findings suggest that obestatin represents a promising therapeutic target for the treatment of metabolic disorders, including diabetes and obesity [91,92]. However, future research could be expanded to investigate how obestatin interacts with the hypothalamic center involved in the regulation of food intake, particularly in the context of diabetes and feeding disorders like obesity. Interestingly, and relevant to the topic of obesity, obestatin appears to have an influence on atherosclerotic cardiovascular disease. It shows that obestatin may have a cardioprotective role, could impact on controlling blood pressure, and also enhances papillary muscle contractility and beta-adrenergic responsiveness, but only in type 1 diabetic rats [93]. Other studies have demonstrated that obestatin administration in rats fed a high-fat diet can protect against non-alcoholic fatty liver disease [94]. More interestingly, obestatin also shows neuroprotective properties and, by improving the function of dopaminergic neurons, it alleviates the progression and symptoms of Parkinson’s disease [95]. Since this peptide demonstrates properties influencing memory retention, vasodilation, and neuronal survival, further studies could be extended to investigate its effects on the neuroendocrine systems as well as the reward system. Interestingly, a therapeutic application for obestatin has also been found in muscle regeneration through the stimulation of satellite stem cells and myofiber hypertrophy [96].

Obestatin has emerged as a multifunctional peptide involved in growth, reproduction, and appetite regulation. However, research has yielded inconsistent results depending on species, administration routes, and experimental conditions. These discrepancies underscore the complexity of obestatin’s function and emphasize the need for further research to fully elucidate its neuroendocrine mechanisms and physiological significance.

## Figures and Tables

**Table 2:** The influence of obestatin on the gonadotrophic axis.

Authors	Research Model	Materials and Methods	Outcome
Mészárosová et al., 2008 [83]	Granulosa cells from Slovakian white gilts	Medium supplemented with obestatin	Obestatin stimulated progesterone secretion to the culture medium but did not affect the secretion of estradiol and testosterone.
Romani et al., 2012 [87]	Human luteal cells	Medium supplemented with obestatin	Obestatin decreased progesterone and prostaglandin E2 and F2α secretions into the medium.
Luque et al., 2014 [88]	Pituitary cells from baboons and mice	Medium supplemented with obestatin	Obestatin did not affect prolactin, LH, and FSH releases.
Wójcik-Gładysz et al., 2019 [89]	Female sheep	Intracerebroventricular infusions	Obestatin increased GnRH mRNA expression in the preoptic area. Obestatin decreased the GnRH mRNA expression in the median eminence, without affecting the level of this peptide mRNA in the anterior hypothalamic area. Central obestatin infusion evoked the restraining of IR GnRH material in the median eminence. Obestatin increased Kiss mRNA and decreased PDyn mRNA expression but not the NKB mRNA in the arcuate nucleus.
Szlis et al., 2020 [90]	Female sheep	Intracerebroventricular infusions	Obestatin decreased LHβ mRNA expression and increased accumulation of LH protein in gonadotrophic cells, and as a result of these changes, the level of LH concentration in peripheral blood also decreased. Moreover, FSHβ mRNA expression and protein accumulation in pituitary cells increased after obestatin treatment, but at the same time, obestatin did not change the pulsatile pattern of FSH release.

## Data Availability

Data sharing is not applicable to this article as no datasets were generated or analyzed during this study.
